# Identifying risk factors and implications for beach drowning prevention amongst an Australian multicultural community

**DOI:** 10.1371/journal.pone.0262175

**Published:** 2022-01-11

**Authors:** Mark Woods, William Koon, Robert W. Brander

**Affiliations:** 1 School of Biological, Earth and Environmental Sciences, UNSW Sydney, Sydney, Australia; 2 UNSW Beach Safety Research Group, UNSW Sydney, Sydney, Australia; Curtin University, AUSTRALIA

## Abstract

Multicultural communities in Australia are recognised as a priority area for drowning prevention, but no evidence-based study has addressed their knowledge of beach safety. This study used an online survey tool to identify and examine risk factors relating to swimming ability, beach visitation characteristics and behaviour, and beach safety knowledge of the Australian Southern Asian community to assist in the development of future beach safety interventions. Data was obtained through 249 online and in-person surveys of people aged > 18 years. Most respondents reported poor swimming ability (80%), often swam in in the absence of lifeguards (77%), did not understand the rip current hazard (58%), but reported that they entered the water (76%) when visiting beaches. Close to one-quarter (28%) had not heard, or didn’t know the purpose, of the red and yellow beach flags, which identify lifeguard supervised areas on Australian beaches. Length of time living in Australia is an important beach safety consideration for this community, with minimal differences in terms of gender and age. Those who have lived < 10 years in Australia visit beaches more frequently and are less likely to have participated in swimming lessons, be able to swim, heard of the flags or swim between them, understand rip currents, or have participated in a beach safety program. Very few (3%) respondents received beach safety information from within their own community. The importance of beach safety education and swimming lessons within the Southern Asian community should be prioritised for new and recent migrants to Australia.

## 1. Introduction

Australia is a coastal nation known for its popular beaches that support a range of recreational activities, but with large waves, strong currents, changing tidal conditions and varied and dynamic morphology, Australian coastal environments can also be dangerous [1–4]. Between 2020–2021 there were 136 coastal drowning deaths in Australia, an increase on the 17-year average of 114 [5]. Beach locations accounted for almost half (48%) of these drownings in 2020–2021 and 45% (n = 874) since 2004 [5]. Beach related drowning is an ongoing concern in Australia, as every drowning is associated with significant societal, economic, and emotional costs [6], and while any beachgoer who enters the water is potentially at risk of drowning, the recent Australian Water Safety Strategy 2030 [7] has identified multicultural communities as being a particular population of risk that deserves further efforts in relation to drowning prevention for a number of reasons.

Australia is a diverse, multicultural country with close to half its population born, or having at least one parent born, overseas and approximately one-fifth do not speak English as their first language [8, 9]. This diversity is reflected in the drowning statistics with people born overseas representing 47% (n = 911) of the entire coastal drowning death toll in Australia between 2004 and 2021 [5]. Multicultural communities in Australia present specific risk factors such as low swimming ability, water safety knowledge, experience, familiarity with the Australian coastal environment, and low levels of hazard awareness and risk perception [7, 9]. Other significant risk factors include varying cultural and religious beliefs and values, both in general and specifically relating to coastal usage. This, as well as language barriers, can make it challenging to educate multicultural communities on beach safety [9, 10]. Collectively, these challenges should be placed in the context of existing attempts at safeguarding beachgoers in Australia as well as existing studies that have attempted to understand beachgoer hazard awareness and behaviour.

### 1.1. Australian beach safety and hazards

Many Australian beaches are patrolled by professional lifeguards and/or volunteer surf lifesavers who designate supervised, safer areas for swimming by installing a pair of red and yellow beach flags near the shoreline. The primary beach safety message in Australia is to ‘swim between the red and yellow flags’ [11]. However, less than 5% of the approximately 11,000 beaches in Australia are patrolled and between 2020–2021, 74% of drowning deaths occurred more than 1 km away from a lifesaving service [5]. Anecdotal evidence suggests that some multicultural communities misinterpret the red and yellow flags as being private swimming areas or avoid them because they associate the red flag as meaning ‘dangerous’ [12–14].

Beach safety signage is also used to make beachgoers aware of potential beach hazards, but several studies have shown the overall effectiveness of signage is poor [15, 16]. Beach safety is also promoted with brochures, online videos, social media, public service announcements and national campaigns [17, 18] as well as community and school-based education [19], but evaluation of these approaches is lacking [20–22]. Furthermore, a key action from the Australian *Addressing Drowning in Multicultural Communities Symposium* in 2018 was to simplify language to improve understanding of key water safety messages, avoid confusion, and encourage community consultation [23]. This ensures that messages are culturally appropriate and translated in the correct context.

The primary physical hazard resulting in fatal beach drowning in Australia are rip currents which result in an average of 21 confirmed fatalities per year [1, 5, 24], including individuals from multicultural communities [25]. This value is more than the average yearly fatalities caused by other Australian natural hazards such as cyclones, floods and bushfires combined [26]. These strong, narrow flows, which extend seaward from the beach through the surf zone and some distance beyond [27] are ubiquitous features on Australian surf beaches, but numerous studies have shown that most beachgoers, both in Australia and overseas, have poor knowledge of rip currents, particularly in respect to identification [28–33].

### 1.2. Multicultural beach safety

A small number of studies have addressed beach safety knowledge of international students and tourists in Australia [13, 32, 34], finding that most arrive with limited beach safety knowledge, but no study has yet to evaluate the issue of migrants and beach safety. A global literature review [35] found that while ethnic-minority populations, including migrants, were reported as being at greater risk of drowning, few studies have addressed drowning among these high-risk populations, including in the Australian context.

Existing research related to drowning amongst Australian migrants has been limited to epidemiological studies [9, 36] or assessments of swimming proficiency and water safety knowledge [12, 37]. Other studies have suggested multicultural communities are at a greater risk of drowning than the general Australian population due to a lack of beach safety education and swimming experience and lower participation in swim education programs [9, 37, 38]. It has been suggested that children of migrants are at increased risk of drowning compared to those of Australian-born parents as migrant parents are less likely to be aware of water safety issues, be able to swim and have CPR training [39, 40]. Other barriers to swimming participation include resources, affordability and modesty [41, 42]. Lack of proficient swimming education and lesser swimming ability amongst people from multicultural communities may contribute to their high-risk status in beach environments [43].

### 1.3. Study aims and objectives

The primary aim of the study is to use a survey tool to examine demographics, swimming ability, beach safety knowledge, and beachgoing behaviour of the Southern Asian community in Australia. This community refers to those born in, or with familial ties, to Afghanistan, Bangladesh, Bhutan, India, Nepal, Pakistan, or Sri Lanka, and was chosen for several reasons. As of 2016 almost 8% (approximately 1.9 million) of Australians were either born in this region or had at least one parent who was [8, 44]. Indians represent the second largest migrant population in Australia, which more than doubled between 2009 and 2020 [45, 46]. These trends are evident in the 2009–2019 drowning statistics, which show that people born in India accounted for the highest proportion of migrants who drowned at Australian beaches [9]. Migrants from Southern Asian countries have accounted for 5.3% (n = 78) of all coastal drowning deaths in Australia since 2004 [5] even though in 2016 they made up only 3.3% of the Australian population [44], which suggests that they are over-represented in the drowning statistics. This community also has a greater English language proficiency compared to other communities [47], an important factor given that the study survey tool was administered in English.

A secondary aim of this study is to compare the Southern Asian community with the overall Australian population in regards to specific beach safety considerations and the study objectives involve assessments of whether gender, age, and time in Australia (TIA) are related to self-reported swimming ability, beach visitation and safety knowledge. The motivation for this study was to provide evidence for the development of culturally appropriate beach safety education programs specific to the Australian Southern Asian community. It is hoped that outcomes of this study can be used to improve beach safety of other multicultural communities, and indeed any beachgoer, in Australia.

## 2. Methods

This study used an online Multicultural Beach Safety (MBS) survey designed using the web-based Qualtrics survey platform [Ver. August 2021]. The MBS survey was restricted to participants aged over 18 years who self-identified as being from a Southern Asian background. Ethics approval was granted for the survey by the UNSW Sydney Human Research Ethics Advisory Panel under project number HC200951.

### 2.1. Survey design

The survey structure and questions were modelled on previous Australian beachgoer surveys [28, 32, 34], then modified for the Southern Asian community through an online workshop held on November 19, 2020. The workshop included representatives from community groups and organisations working with the Southern Asian community in Australia. Following two qualifying questions related to being over 18 years of age and from a Southern Asian community, the MBS survey consisted of a maximum of 38 questions arranged across four thematic sections: i) demographics; ii) beachgoing experience and swimming ability; iii) beach safety and hazards, and iv) beach safety education. Questions were a mix of multiple-choice, Likert Scale, and photographic based heatmap questions. The median time taken to complete the online survey was 8 minutes and 44 seconds. A copy of the survey is provided in S1 Survey.

### 2.2. Survey distribution

The MBS survey was made available online via https://tiny.cc/beachsurvey and a QR code linked to the survey was also created. Surveys were disseminated over three months from February 18^th^ to May 7^th^, 2021, using a variety of methods. The survey was promoted and shared with the assistance of several Australian Non-Government Organisations (NGOs) who work with the Southern Asian community through social media and organisational contacts. Additionally, a Facebook page called the *UNSW Multicultural Beach Safety Survey* was created. A total of 72 Southern Asian community groups were contacted by email and provided with information about the purpose of the survey and a request to share the link, with a response rate of 17% (n = 12). Similarly, 30 Southern Asian related Facebook community groups were contacted and 12 shared the survey (response rate 37%). Posts were created on the survey Facebook page and were requested to be shared on these group pages on February 19^th^, March 31^st^, April 19^th,^ and May 3^rd^, 2021.

In-person surveys were conducted at the Sri Venkateswara Temple (SVT) in Helensburgh, NSW, near Sydney. The SVT is the largest Hindu Temple in NSW and attracts visitors from Southern Asian communities throughout Australia. Permission to conduct the surveys was granted by SVT leadership for three weekends in March and April 2021. On each occasion, two volunteers attended the temple and approached potential participants on the temple grounds. Respondents were asked to fill out the survey on a tablet provided to them, scan a QR code and fill it out on their mobile device, or take a QR code flyer to fill out the survey later. For this reason, it is not possible to determine the total amount of surveys completed by visitors to the SVT Temple, or the response rate, although 127 refusals were recorded. Of note, the SVT is located only several kilometres from Stanwell Park Beach, which is rated as highly hazardous due to strong wave and rip current action [48] and many SVT visitors also visit Stanwell Park Beach after visiting the Temple. In September 2018, an international student from India drowned in a rip current at Stanwell Park while swimming outside of the flagged area [25].

### 2.3. Data analysis

Survey responses were exported from Qualtrics to Microsoft Excel [Ver. 16.52] for reformatting before being imported to R Studio Version [Ver. 1.4.1717] for analysis. The final sample of 249 surveys had at least 29% of questions completed and had to include answers to the first section of the survey consisting of eight demographic questions and three beach visitation questions. Descriptive statistics were calculated to describe the sample, and several different regression models were used to evaluate specific outcome variables of interest. Multiple logistic regression was used to evaluate if age, gender or time in Australia (TIA) predicted ability to swim, confidence swimming in waves, participation in swimming lessons, if the respondent had heard of the red and yellow flags or (separately) knew their meaning, if the respondent would enter the water with no flags, and participation in any sort of beach safety program. Multiple ordinal regression was used to evaluated if age, gender or time in Australia (TIA) predicted frequency of beach visits, entering the water, swimming while fully clothed, looking for hazards, swimming between the flags and ability to spot rip currents in a picture. An alpha level of 0.05 was used for all tests.

Predictors age and TIA were both categorical questions with ranges in the survey, and converted to continuous predictor variables preserving the ordinal ranks [49] where age groups and TIA categories were labelled in sequence from lowest to highest (0, 1, 2, 3, 4, 5). While the categories for age and TIA may not have perfectly equal spacing, Pasta [50] and Robitzsch [49] specify that they can be treated as continuous in almost all cases, even when spacing is not equal across categories. This was done to assess for a trend in the predictor variables age, gender, and TIA for each of the outcome variables.

## 3. Results

This section summarises results of the Multicultural Beach Safety (MBS) survey and presents data separated by the three primary variables relating to the survey respondents: age, gender and (residence) time in Australia (TIA). A total of 249 unique responses were received for the survey, 230 of which were fully complete. Sample sizes varied for some variables due to the skip logic structure of some of the survey questions. If not referred to by Figure or Table, reported data can be found in S1 Data.

### 3.1. Demographics

**Fig 1:**
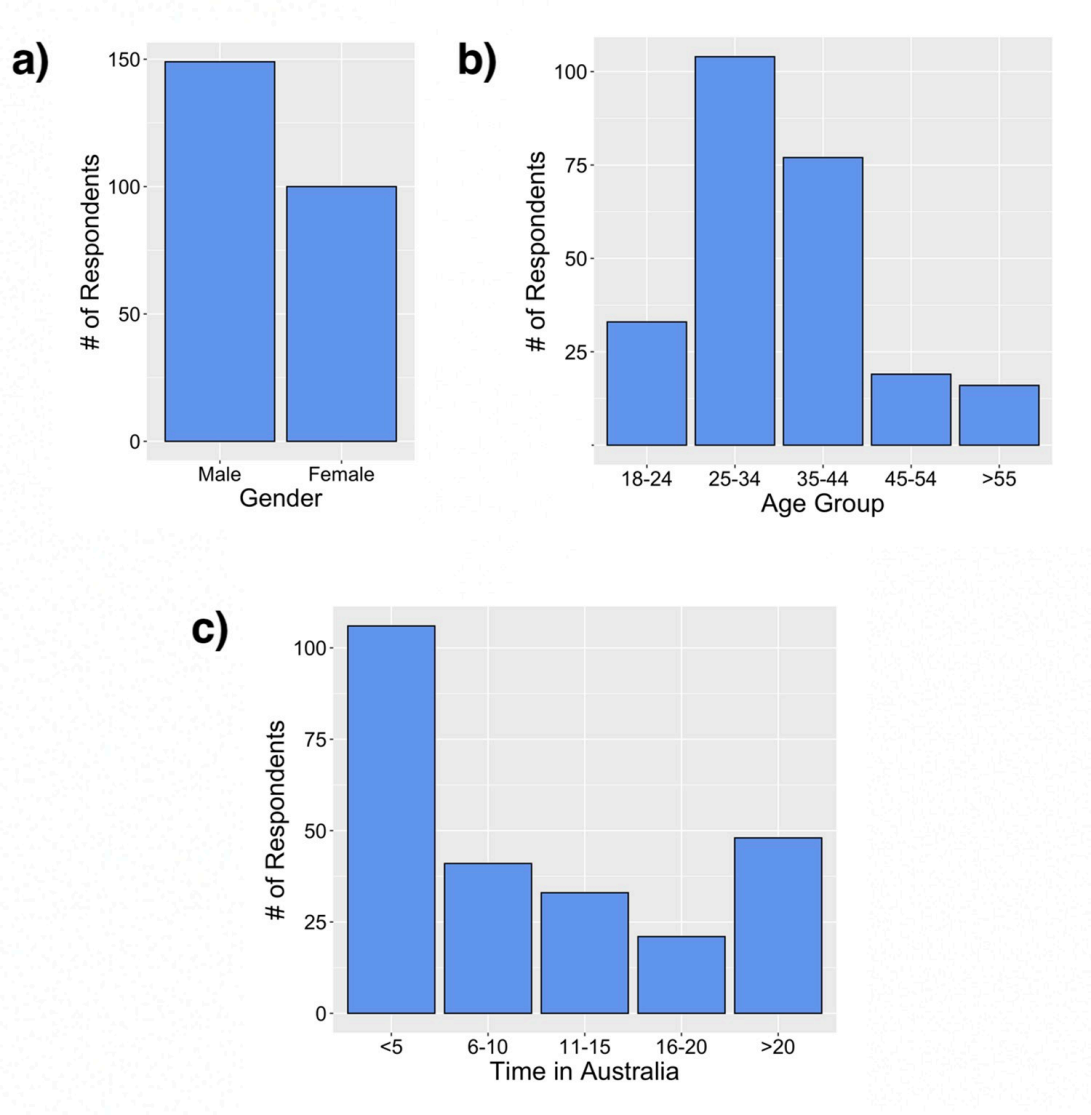
Survey participants by: a) gender; b) age group; and c) time in Australia in years. Sample size is n = 249 for each.

Survey respondents were predominantly from major cities, particularly Sydney, NSW (63%). Most were male (Fig 1A), aged 24–44 years (73%; Fig 1B), and almost half (46%) indicated they had lived in Australia for less than 5 years (Fig 1C). Most respondents identified with the Indian community (60%) followed by the Nepalese community (18.5%) and most (89%) were born outside of Australia, with just over half born in India (53%; Table 1). The majority (89%) spoke English and at least one other language (Table 1).

**Table 1:** Cultural and language demographics of survey respondents by gender, age group and time in Australia.

	Gender %		Age Group % (years)					Time in Australia % (years)				
	M	F	18–24	25–34	35–44	45–54	> 55	< 5	6–10	11–15	15–20	> 20
**Multicultural community (n = 249)**												
Indian (60.2%)	59.1	62.0	45.5	52.9	74.0	73.7	56.3	55.7	68.3	69.7	85.7	45.8
Nepalese (18.5%)	20.8	15.0	39.4	24.0	7.8	0.0	12.5	30.2	14.6	15.2	0.0	6.3
Other (21.3%)	20.1	23.0	15.2	23.1	18.2	26.3	31.3	14.2	17.1	15.2	14.3	47.9
**Country of birth (n = 249)**												
India (52.6%)	53.0	52.0	39.4	47.1	66.2	57.9	43.8	53.8	63.4	66.7	71.4	22.9
Nepal (18.1%)	20.1	15.0	36.4	24.0	7.8	0.0	12.5	29.2	14.6	15.2	0.0	6.3
Australia (4.4%)	3.4	6.0	9.1	4.8	1.3	5.3	6.3	0.0	2.4	0.0	0.0	20.8
Other (24.9%)	23.5	27.0	15.2	24.0	24.7	36.8	37.5	17.0	19.5	18.2	28.6	50.0
**Religion (n = 249)**												
Hinduism (63.1%)	66.4	58.0	51.5	60.6	67.5	63.2	81.3	66.0	73.2	66.7	57.1	47.9
Islam (14.5%)	12.1	18.0	15.2	17.3	11.7	21.1	0.0	6.6	12.2	15.2	19.0	31.3
Other (22.5%)	21.5	24.0	33.3	22.1	20.8	15.8	18.8	27.4	14.6	18.2	23.8	20.8
**Languages (n = 249)**												
English only (11.2%)	8.1	16.0	15.2	10.6	10.4	5.3	18.8	7.5	7.3	15.2	9.5	20.8
English + at least one other (88.8%)	91.9	84.0	84.8	89.4	89.6	94.7	81.3	92.5	92.7	84.8	90.5	79.2

Values reported are percentages of the total sample size (n = 249).

### 3.2. Beach visitation and swimming ability

**Fig 2:**
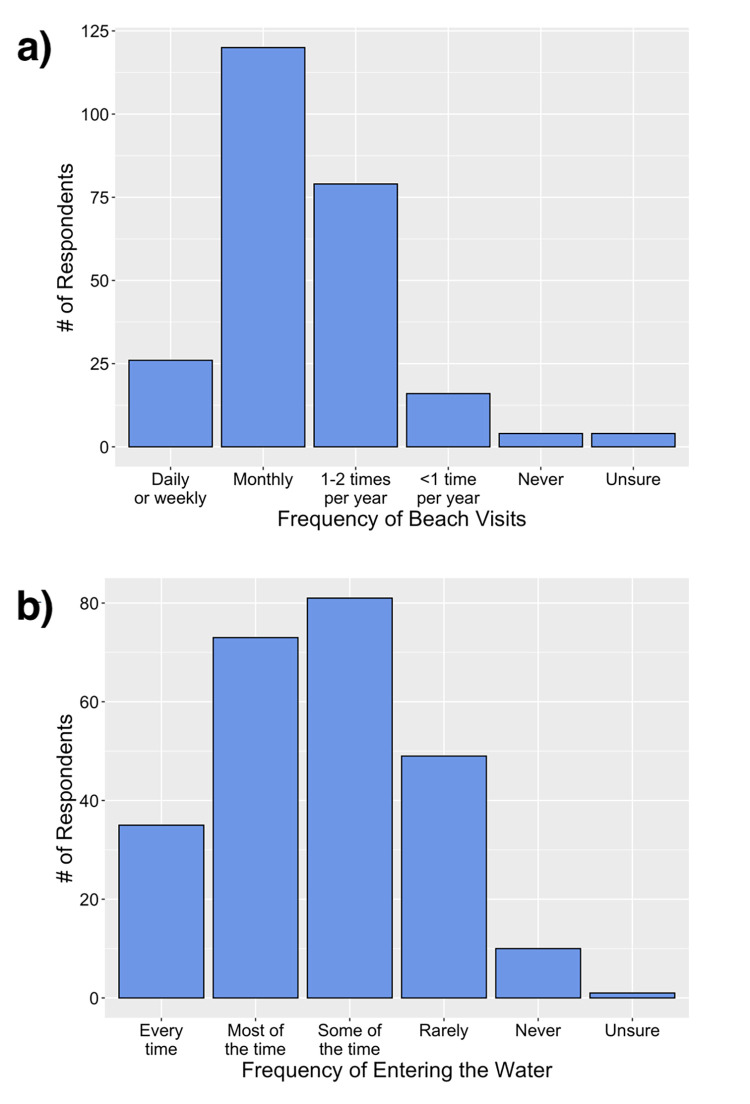
Responses of surveyed participants in terms of: a) frequency of beach visits; and b) frequency of entering the water when they visit a beach. Sample size is n = 249 for both.

Most respondents were infrequent beachgoers, visiting beaches approximately once a month (48%) or 1–2 times per year or less (40%; Fig 2). While no statistical relationships were identified between beach visitation frequency and gender or age, longer residence in Australia was associated with lower beach visit frequency (Table 2). For each increase in the TIA group, the odds of being more likely to visit the beach is 16% lower, holding age and gender constant (Table 2).

**Table 2:** Summary results for the logistic and ordinal regressions of selected survey response variables.

Response Variable	Type of Regression	Results	Predictor Variables		
			Gender	Age groups (trend)	TIA (trend)
Beach Visit Frequency (n = 249)	Ordinal	*p*-value	.43	.18	.047 *
		Trend OR	0.82	0.83	0.84
		95% CI	0.50–1.33	0.63–1.09	0.71–1.00
Entering Water Frequency (n = 249)	Ordinal	*p*-value	.21	< .001 ***	0.11
		Trend OR	1.35	0.61	1.14
		95% CI	0.85–2.16	0.47–0.79	0.97–1.36
Can you Swim? (n = 249)	Logistic	*p*-value	.01 **	.001 ***	< .001 ***
		Trend OR	2.03	0.59	1.43
		95% CI	1.16–3.59	0.42–0.81	1.16–1.78
Swimming Confidence in Waves (n = 107)	Logistic	*p*-value	.57	.98	.79
		Trend OR	0.76	1.01	0.96
		95% CI	0.50–3.40	0.59–1.68	0.77–1.43
Fully Clothed Frequency (n = 107)	Ordinal	*p*-value	.43	.43	.50
		Trend OR	0.74	0.85	0.92
		95% CI	0.34–1.59	0.57–1.27	0.73–1.17
Swimming Lessons Participation (n = 107)	Logistic	*p*-value	.14	.86	< .001 ***
		Trend OR	0.66	0.97	1.44
		95% CI	0.38–1.15	0.72–1.31	1.19–1.77
Looking for Beach Dangers (n = 107)	Ordinal	*p*-value	.13	.65	.15
		Trend OR	1.69	0.92	1.19
		95% CI	0.86–3.31	0.65–1.31	0.94–1.51
Heard of Flags (n = 240)	Logistic	*p*-value	.78	.15	.003 **
		Trend OR	0.89	0.7	1.77
		95% CI	0.37–2.02	0.43–1.14	1.24–2.68
Meaning of Flags (n = 208)	Logistic	*p*-value	.14	.41	.01 **
		Trend OR	0.51	1.27	0.58
		95% CI	0.20–1.26	0.72–2.28	0.37–0.85
Swim between Flags (n = 208)	Ordinal	*p*-value	.22	.44	< .001 ***
		Trend OR	1.4	0.87	1.39
		95% CI	0.82–2.40	0.68–1.19	1.16–1.68
Enter with No Flags (n = 182)	Logistic	*p*-value	.1	.19	.03 *
		Trend OR	1.78	0.78	1.32
		95% CI	0.89–3.57	0.53–1.13	1.04–1.7
Rip Spotting in Images (n = 84)	Ordinal	*p*-value	0.3	0.27	.01 **
		Trend OR	1.58	0.8	1.46
		95% CI	0.67–3.82	0.54–1.18	1.10–1.98
Safety Program Participation (n = 230)	Logistic	*p*-value	.5	.02 *	.003 **
		Trend OR	0.8	0.66	1.4
		95% CI	0.42–1.54	0.46–0.94	1.12–1.76

TIA = time in Australia; OR = odds ratio; CI = confidence interval. Bold values highlighted with an asterisk indicate statistically significant relationships. Shaded areas were found to be statistically significant. Asterisks represent the significance level of the relationship, where

* = p < 0.05

** = p ≤ 0.01 and

*** = p ≤ 0.001.

**Fig 3:**
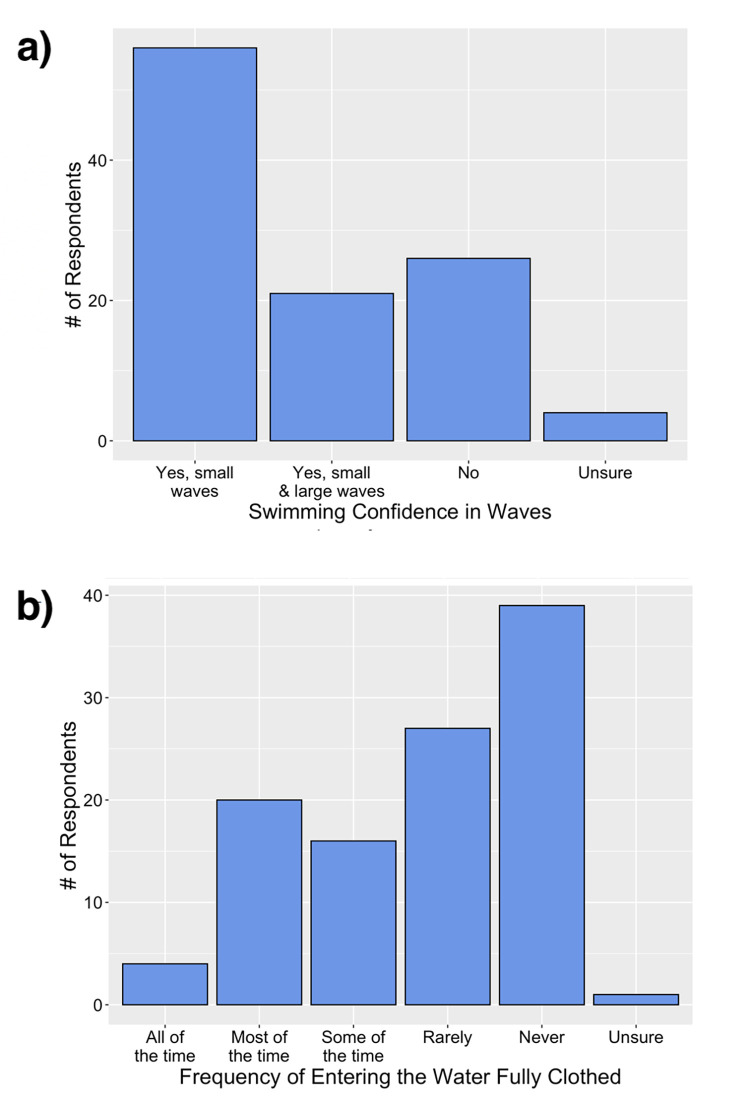
Survey respondents swimming confidence in breaking waves (a) and frequency of entering the water fully clothed (b). Sample size was n = 107 for both survey questions.

Nearly half the respondents reported entering the water either most of the time or every time they visited the beach (30% and 14%, respectively) and 20% indicated they rarely enter the water (Fig 3). There is statistical evidence that older age groups enter the water less frequently. For every increase in age group, the odds of being more likely to enter the water decreases by 39%, holding gender and TIA constant (Table 2). No significant relationship was found between the frequency of entering the water and either gender or TIA. The most frequently cited activities at the beach were playing in the water (73%), picnic/parties/family events (55%), walking/jogging (50%), and swimming (39%).

Almost half the respondents (43%; n = 106) indicated they could not swim (Table 3). Statistically significant relationships were found between swimming ability and respondent gender, age, and TIA. Controlling for the other variables, older respondents were less likely to self-report swimming ability, males were more likely than females to report swimming abilities, and those who had lived in Australia for longer periods were more likely to report higher levels of swimming ability (Table 2). Of those respondents who could swim or were unsure (57.4%), almost half (44%) indicated they could not swim 25 m in a swimming pool without stopping, and 27% thought they could not swim more than 100 m (Table 3). Despite mostly poor swimming skills, 74% of respondents indicated they had previously swum or bathed in the ocean. When asked to rate their swimming ability in the ocean, more than half (54%) indicated they could not swim 25 m without stopping and 19% could not swim more than 100 m (Table 3). Approximately half of the respondents of the subgroup who had previously swum/bathed in the ocean (52%) were only confident swimming in small waves and a further 24% were not confident in waves at all (Fig 3A). The majority of the ocean swimming/bathing subgroup (63%) also indicated they had previously entered the water fully clothed (Fig 3B). However, no statistically significant relationships were found between age, gender, TIA and swimming confidence in waves or entering the water fully clothed (Table 2).

**Table 3:** Swimming ability and swimming lesson history of survey respondents by gender, age group and time in Australia.

	Gender		Age Group (years)					Time in Australia (years)				
	M	F	18–24	25–34	35–44	45–54	> 55	< 5	6–10	11–15	15–20	> 20
**Can you swim? (n = 249)**												
Yes (51.4%)	55.0	46.0	60.6	51.9	49.4	42.1	50.0	47.2	46.3	42.4	57.1	68.8
No (42.6%)	37.6	50.0	30.3	40.4	45.5	57.9	50.0	43.4	51.2	48.5	42.9	29.2
Unsure (6.0%)	7.4	4.0	9.1	7.7	5.2	0.0	0.0	9.4	2.4	9.1	0.0	2.1
**Self-estimate of swimming distance in a swimming pool without stopping (n = 143)**												
< 25 m (43.4%)	40.9	48.0	26.1	50.0	45.2	37.5	37.5	53.3	15.0	47.1	25.0	47.1
Up to 100 m (27.3%)	30.1	22.0	34.8	25.8	31.0	12.5	12.5	23.3	55.0	29.4	8.3	23.5
> 100 m (10.5%)	9.7	12.0	17.4	6.5	11.9	12.5	12.5	8.3	5.0	5.9	33.3	11.8
> 500 m (9.1%)	9.7	8.0	13.0	3.2	4.8	37.5	37.5	3.3	15.0	5.9	8.3	17.6
Unsure (9.8%)	9.7	10.0	8.7	14.5	7.1	0.0	0.0	11.7	10.0	11.8	25.0	0.0
**Previously swam/bathed in the ocean (n = 143)**												
Yes (74.1%)	72.0	78.0	65.2	80.6	66.7	75.0	87.5	63.3	85.0	70.6	83.3	85.3
No (25.2%)	26.9	22.0	34.8	17.7	33.3	25.0	12.5	35.0	15.0	29.4	16.7	14.7
Unsure (0.7%)	1.1	0.0	0.0	1.6	0.0	0.0	0.0	1.7	0.0	0.0	0.0	0.0
**Self-estimate of swimming distance in the ocean without stopping (n = 107)**												
< 25 m (54.2%)	52.9	56.4	40.0	60.8	53.6	50.0	42.9	56.4	52.9	50.0	30.0	62.1
Up to 100 m (18.7%)	19.1	17.9	33.3	11.8	32.1	0.0	0.0	23.1	11.8	25.0	40.0	6.9
> 100 m (5.6%)	5.9	5.1	6.7	3.9	3.6	0.0	28.6	0.0	5.9	8.3	0.0	13.8
> 500 m (9.3%)	11.8	5.1	6.7	3.9	7.1	50.0	28.6	0.0	17.6	8.3	10.0	17.2
Unsure (12.1%)	10.3	15.4	13.3	19.6	3.6	0.0	0.0	20.5	11.8	8.3	20.0	0.0
**Participated in swimming lessons (n = 249)**												
Yes (43.0%)	38.3	50.0	36.4	40.4	42.9	57.9	56.3	32.1	36.6	39.4	57.1	68.8
No (55.8%)	59.7	50.0	63.6	56.7	57.1	42.1	43.8	66.0	61.0	60.6	42.9	31.3
Unsure (1.2%)	2.0	0.0	0.0	2.9	0.0	0.0	0.0	1.9	2.4	0.0	0.0	0.0
**Reasons for no previous swimming lessons (n = 142)** [multiple answers allowed]												
Lack of time (39.4%)	41.3	36.0	42.9	35.5	45.5	50.0	14.3	44.3	42.3	40.0	22.2	26.7
Financial cost (23.9%)	21.7	28.0	23.8	25.8	22.7	37.5	0.0	20.0	34.6	25.0	44.4	13.3
Lack of availability (36.6%)	35.9	38.0	52.4	30.6	43.2	25.0	14.3	45.7	23.1	25.0	44.4	33.3
Other (25.4%)	23.9	28.0	23.8	32.3	13.6	0.0	71.4	14.3	38.5	30.0	33.3	46.7
**Location of previous swimming lessons (n = 101)** [multiple answers allowed]												
Australia (68.3%)	58.5	79.2	66.7	67.5	65.6	70.0	75.0	54.5	60.0	69.2	63.6	81.3
Other country (36.6%)	45.3	27.1	33.3	35.0	43.8	30.0	25.0	45.5	46.7	38.5	54.5	12.5
Unsure (1.0%)	1.9	0.0	0.0	2.5	0.0	0.0	0.0	3.0	0.0	0.0	0.0	0.0
**Age of previous swimming lessons (n = 104)** [multiple answers allowed]												
Child < 12 y (35.5%)	34.5	38.8	50.0	36.6	40.6	20.0	22.2	21.2	46.7	15.4	36.4	56.3
Teen 13–17 y (16.8%)	16.4	18.4	41.7	17.1	9.4	20.0	11.1	18.2	6.7	7.7	18.2	25.0
Adult > 18 y (54.2%)	56.4	55.1	16.7	61.0	62.5	60.0	55.6	66.7	53.3	76.9	81.8	28.1
Unsure (1.9%)	1.8	2.0	8.3	0.0	0.0	0.0	11.1	3.0	0.0	0.0	0.0	3.1

Values reported are percentages of sample size. Sample size varied due to the skip logic structure of the survey questions.

More than half the respondents (56%) indicated they had not previously had organised/formal swimming lessons (Table 3). Of those who had, most (68%) had lessons in Australia, and half (50%) had lessons as an adult (>18 years of age; Table 3). Lack of time (40%), availability (37%) and financial costs (24%) were all common reasons given as to why many (56%) had not previously had swimming lessons (Table 3). Other reasons (26%) included being self-taught, taught by a family member or that it was not important for them either culturally or personally. While no relationships were identified between swimming lesson participation and gender or age, more time in Australia was associated with higher odds of self-reported swimming lesson participation (Table 2).

### 3.3. Beach safety behaviour

Respondents identified the most important factors related to visiting an ocean beach to them as clean beach and water (95%), the presence of lifeguards/lifesavers (81%), the beach being safe for swimming (79%), easy parking (73%), calm water (73%) and space for large picnics and gatherings (65%). All respondents were shown two images of a beach with prominent rip currents appearing as darker, seemingly calmer, gaps amongst breaking waves and whitewater (S1 Survey) and were asked to select the location in each photo where they would enter the water to swim, wade or play. Many respondents (37%) made the potentially dangerous choice of choosing to enter the water in front of a rip current in one of the two images.

Most respondents (52%) indicated they check for dangers when visiting the beach either all the time or most of the time (40% and 32%, respectively) and 16% indicated they rarely, or never, check for dangers (Table 4). No statistically significant relationships were identified between gender, age or TIA and looking for beach dangers (Table 2). Respondents were worried about water hazards such as drowning (27%), rip currents (18%) and waves (16%), more than environmental/biological hazards such as sunburn (12%) or sharks (8%; Table 4).

**Table 4:** Beach safety behaviour and knowledge of respondents by gender, age group and time in Australia.

	Gender		Age Groups (years)					Time in Australia (years)				
	M	F	18–24	25–34	35–44	45–54	> 55	< 5	6–10	11–15	15–20	> 20
**Frequency of looking for dangers at the beach (n = 240)**												
All the time (39.6%)	36.8	43.8	51.5	40.8	29.3	55.6	37.5	40.0	48.8	30.3	42.1	36.2
Most of the time (31.7%)	35.4	26.0	27.3	31.6	34.7	27.8	31.3	29.0	31.7	30.3	31.6	38.3
Some of the time (12.1%)	11.1	13.5	12.1	8.2	16.0	16.7	12.5	12.0	4.9	24.2	10.5	10.6
Rarely (8.8%)	11.1	5.2	3.0	8.2	13.3	0.0	12.5	8.0	9.8	12.1	10.5	6.4
Never (7.1%)	4.9	10.4	6.1	11.2	4.0	0.0	6.3	10.0	4.9	3.0	5.3	6.4
Unsure (0.8%)	0.7	1.0	0.0	0.0	2.7	0.0	0.0	1.0	0.0	0.0	0.0	2.1
**Greatest worry when visiting the beach (n = 240)**												
Drowning (26.7%)	27.1	26.0	30.3	26.5	24.0	38.9	18.8	25.0	34.1	27.3	26.3	23.4
Rip currents (17.9%)	19.4	15.6	18.2	18.4	18.7	16.7	12.5	15.0	9.8	15.2	31.6	27.7
Waves (16.3%)	15.3	17.7	24.2	14.3	16.0	11.1	18.8	22.0	7.3	21.2	10.5	10.6
Sunburn (12.1%)	9.0	16.7	9.1	12.2	12.0	16.7	12.5	12.0	19.5	9.1	5.3	10.6
Sharks (7.9%)	9.7	5.2	3.0	10.2	5.3	5.6	18.8	7.0	14.6	9.1	0.0	6.4
Other (17.9%)	18.1	17.7	12.1	17.3	22.7	11.1	18.8	16.0	14.6	18.2	26.3	21.3
Unsure (1.3%)	1.4	1.0	3.0	1.0	1.3	0.0	0.0	3.0	0.0	0.0	0.0	0.0
**Frequency of swimming between flags (n = 208)**												
All the time (28.5%)	25.8	32.1	27.6	19.8	32.3	52.9	35.7	18.1	29.4	21.4	47.1	43.5
Most of the time (23.1%)	29.0	14.3	13.8	26.7	24.2	23.5	14.3	19.3	29.4	21.4	11.8	30.4
Some of the time (7.7%)	9.7	4.8	17.2	8.1	3.2	5.9	7.1	8.4	5.9	10.7	5.9	6.5
Rarely (10.6%)	10.5	10.7	13.8	14.0	4.8	5.9	14.3	16.9	0.0	7.1	11.8	8.7
Never (12.5%)	10.5	15.5	13.8	10.5	19.4	0.0	7.1	15.7	14.7	21.4	11.8	0.0
I don’t swim at the beach (12.0%)	9.7	15.5	10.3	10.5	12.9	11.8	21.4	15.7	8.8	10.7	5.9	10.9
Unsure (5.8%)	4.8	7.1	3.4	10.5	3.2	0.0	0.0	6.0	11.8	7.1	5.9	0.0
**Entered the water at a beach without flags (n = 182)**												
Yes, mostly by myself (14.3%)	19.8	5.6	4.0	18.2	16.7	6.7	9.1	14.5	22.6	20.0	0.0	9.8
Yes, mostly with family or friends (39.0%)	37.8	40.8	40.0	40.3	33.3	60.0	27.3	24.6	54.8	24.0	62.5	51.2
No (33.5%)	30.6	38.0	44.0	29.9	31.5	33.3	45.5	43.5	16.1	48.0	18.8	26.8
Unsure (13.2%)	11.7	15.5	12.0	11.7	18.5	0.0	18.2	17.4	6.5	8.0	18.8	12.2
**Heard/know of a rip current (n = 237)**												
Heard & know meaning (42.2%)	39.9	45.7	53.1	35.7	39.7	55.6	56.3	26.3	46.3	31.3	61.1	72.3
Heard but don’t know meaning (24.9%)	25.2	24.5	18.8	26.5	26.0	33.3	12.5	30.3	14.6	37.5	27.8	12.8
Never heard (23.6%)	24.5	22.3	21.9	24.5	27.4	5.6	25.0	29.3	31.7	25.0	5.6	10.6
Unsure (9.3%)	10.5	7.4	6.3	13.3	6.8	5.6	6.3	14.1	7.3	6.3	5.6	4.3

Values reported are percentages of the sample size which varied depending on the question.

Most respondents (83%) had heard of the red and yellow flags on Australian beaches, but 13% had not and 5% were unsure. Of those who had heard of the flags, 11% incorrectly identified their meaning and a further 6% were unsure, suggesting that just over a quarter of the respondents (27%) did not know of, or did not have a good understanding of, the red and yellow flags. While no statistical relationships were identified between gender or age and if someone had heard of the flags or understood their meaning, those with more time in Australia were more likely to have heard of the flags (OR = 1.77), but less likely to correctly identify their meaning (OR = 0.58; Table 2).

Of those respondents who had heard of the red and yellow flags, half (54%) indicated they do not always swim between them, and 23% indicated they rarely or never swim between them (Table 4). When asked if they had ever entered the water to swim at a beach with no flags, 53% indicated they had, mostly as a family or group (39%; Table 4). Again, TIA was the only statistically significant predictor of swimming between the flags and having previously entered the water to swim at an unpatrolled beach. Controlling for age and gender, more time in Australia indicated higher likelihood of swimming between the flags (OR = 1.39) and previously entered the water at unpatrolled beaches (OR = 1.32; Table 2).

**Fig 4:**
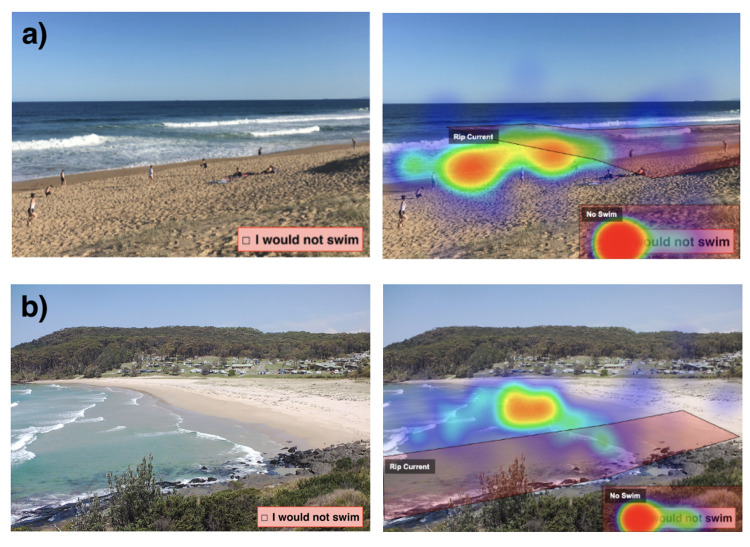
Images shown to respondents who indicated they knew, or had heard of, rip currents asking them if they could identify a rip current in each image. Left panel shows original images. Right panel shows a heat map of respondent choices of where they believed a rip current to be with hot colours (red) indicating more responses. Both images contain a prominent rip current evident as the dark gap between white water. Both rip areas are defined by polygons. Photos courtesy of R. Brander.

A quarter of the respondents had never heard of a rip current (25%), and another quarter (24%) had, but didn’t know what they are (Table 4). Those who indicated they knew what a rip current was (42%) or were unsure (9%), were then asked to identify the location of a rip current in two separate images (Fig 4). In the first (Fig 4A), most respondents (68%) did not identify the rip, with many incorrectly selecting ‘No Rip’. In the second photo (Fig 4B) 56% correctly chose the rip current location, which showed the rip current from a more oblique angle of view. Those with more time in Australia were more likely to identify the correct rip current location in the photos (Table 2).

### 3.4. Access to beach safety information

A quarter of respondents (24%) had previously attended a beach or water safety education program (Table 5). Statistically significant relationships existed between respondent age and TIA and participation in a beach/water safety program. Controlling for other variables, older respondents were less likely, and those with more time in Australia were more likely, to have participated in a safety program (OR = 0.66 and 1.4, respectively, Table 2).

**Table 5:** Survey respondent experience and opinions relating to beach safety education by gender, age group and time in Australia.

	Gender		Age Groups (years)					Time in Australia (years)				
	M	F	18–24	25–34	35–44	45–54	> 55	< 5	6–10	11–15	15–20	> 20
**Attended a beach/water safety education program (n = 230)**												
Yes (23.9%)	28.9	20.7	33.3	22.9	26.8	17.6	12.5	19.8	17.5	26.7	22.2	37.0
No (75.2%)	71	77.9	66.7	77.1	73.2	82.4	81.3	80.2	80	73.3	77.8	60.9
Unsure (0.9%)	0.0	1.4	0.0	1.0	0.0	0.0	6.3	0.0	2.5	0.0	0.0	2.2
**Desired amount of beach safety information (n = 230)**												
Present amount good (20.4%)	24.1	14.6	13.8	23.7	12.7	11.8	56.3	25.3	30.0	6.5	0.0	19.6
Present amount not good, need more (58.3%)	53.2	66.3	86.2	52.6	62.0	64.7	18.8	53.7	52.5	64.5	77.8	60.9
Beach safety info not important (5.2%)	6.4	3.4	0.0	5.2	7.0	5.9	6.3	5.3	2.5	6.5	5.6	6.5
Unsure (16.1%)	16.3	15.7	0.0	18.6	18.3	17.6	18.8	15.8	15.0	22.6	16.7	13.0
**Translation of beach safety information (n = 230)**												
Yes, need more translation (84.8%)	83.7	86.5	86.2	86.6	78.9	94.1	87.5	83.2	82.5	87.1	83.3	89.1
No (11.7%)	12.8	10.1	13.8	11.3	14.1	0.0	12.5	13.7	17.5	3.2	11.1	8.7
Unsure (3.5%)	3.5	3.4	0.0	2.1	7.0	5.9	0.0	3.2	0.0	9.7	5.6	2.2

Values reported are percentages of sample size.

**Fig 5:**
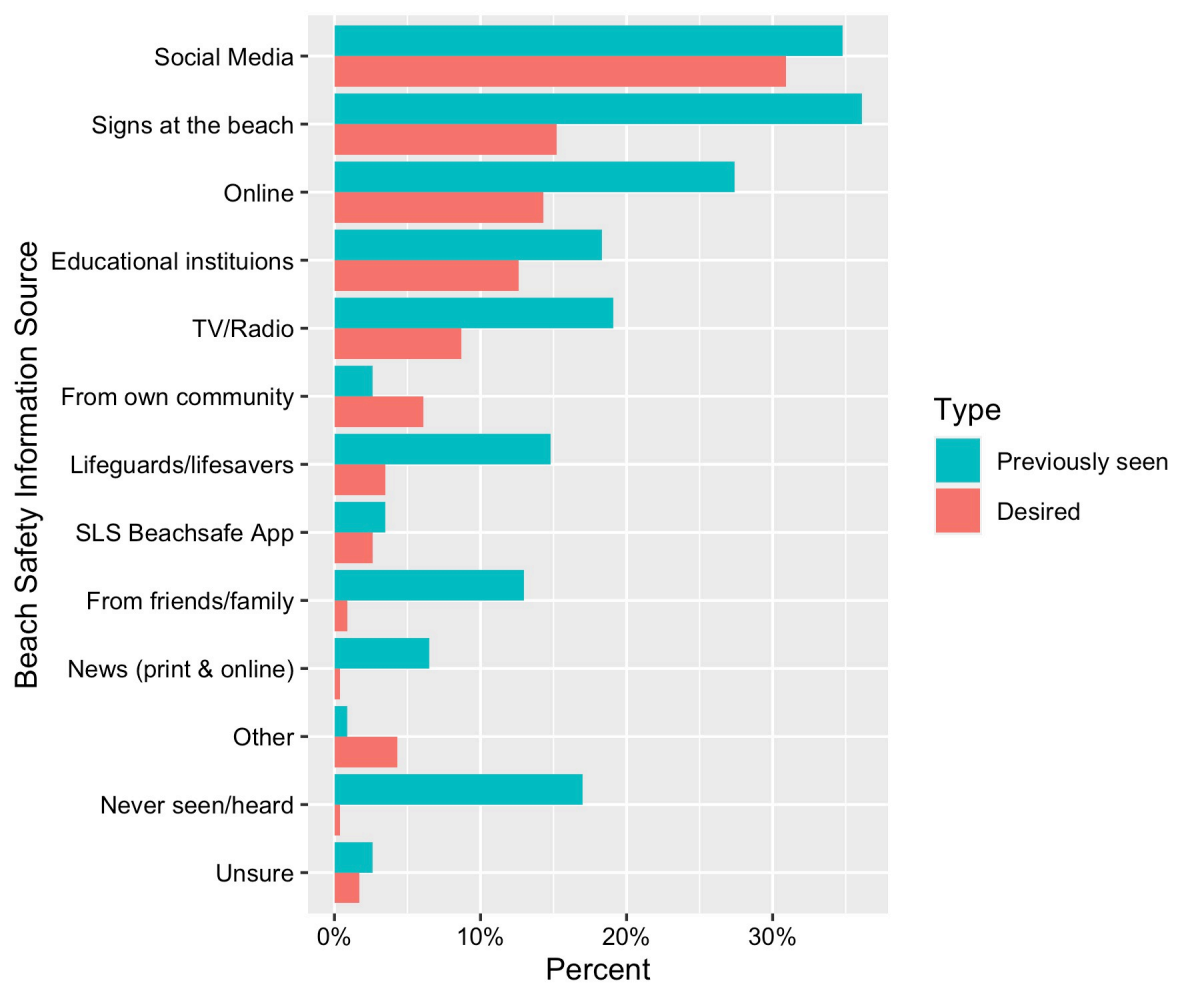
Previous exposure to beach safety information of survey respondents by source (blue bars) and how they would like to receive beach safety information in the future (red bars). Respondents were able to choose multiple answers for both.

The most frequent sources of beach safety information were beach signage, social media, and online web sources (Fig 5). Nearly one-fifth (17%) indicated they had never seen or heard beach safety information before. Almost 60% of respondents thought the existing amount of beach safety information reaching themselves and their community was either not good or needs to be increased (Table 5). The majority (85%; n = 195) felt that beach safety information would be more effective if translated into multiple languages.

## 4. Discussion

The recent Australian Water Safety Strategy [7] identified ’Multicultural Communities’ as a priority area for drowning prevention and called for more research to investigate risk factors for drowning among people from multicultural communities. This is largely due to a paucity of information in this area as also identified by Willcox-Pidgeon et al. [35]. This study represents the first attempt to assess beach safety among multicultural communities, both in Australia and globally. In general the survey respondent distribution was representative of the Australian Southern Asian population with 60% of respondents being from the Indian community compared to 59% of the overall Indian population within the Southern Asian community [44]. However, respondents from the Sri Lankan (5%) and Pakistani (2%) communities were slightly under-represented (14% and 8% of Southern Asian population respectively) while the Nepalese community was over-represented (18% of survey compared to 7% of population [44]).

The results identified an Australian multicultural community who are mostly infrequent beachgoers, non- or poor swimmers, lacking formal swimming lessons and not confident in ocean wave conditions. Yet members of this community do visit a beach, and most enter the water, often fully clothed. Furthermore, many frequently swim outside of the flags, or at unpatrolled beaches, and have poor knowledge of the rip current hazard. Taken as a whole, these results identify several focus areas and implications for future beach safety education and drowning prevention efforts for this particular multicultural community.

A key study objective was to assess whether gender, age, or time in Australia (TIA) help explain variability in swimming ability, beach visitation habits and beach safety knowledge for the Southern Asian community in Australia. Results show that the main determinant affecting these factors is TIA, with minimal significant differences in terms of gender and age (Table 2). Those who have lived in Australia for shorter time periods are more likely to visit the beach, but are less likely to be able to swim, have participated in swimming lessons, heard of the flags, swim between the flags, and be able to spot rip currents (Table 2). The clear implication here is that new and recent migrants to Australia should be a central focus for learn to swim programs and beach safety education.

Previous studies have found strong links between poor swimming ability and lack of beach safety knowledge and a higher risk of drowning [11, 51, 52]. Most of the surveyed Southern Asian community had a poor swimming ability (Table 3) suggesting that adult swimming lessons should be prioritised among this community. This is reinforced by Willcox-Pidgeon et al. [53] who found that adults often miss out on swimming lessons, which generally prioritise children, and by Moran and Wilcox [43] who reported that many new migrant adults do not consider swimming and water safety important for themselves but do prioritise swimming lessons for their children. The results of this study, therefore, support the call to action outlined by Scarr et al. [23] to promote the benefits of learning swimming and water safety skills amongst multicultural communities and to assess the effectiveness of such programs [38, 41, 53].

Beasely Intercultural [42] noted in a qualitative study that many migrants knew someone who had drowned, either in Australia or their home country, and were therefore cautious around water. This is reflected in the results of this study where most of the surveyed Southern Asian community were beach safety aware, with almost three quarters indicating they check for dangers at the beach either all, or most, of the time (Table 4) and most believing lifeguards/lifesaving services are important. However, of concern is that almost a quarter either had not heard of the red and yellow beach safety flags or did not understand their meaning (Table 2). While anecdotal information suggests that some multicultural communities avoid flagged and supervised areas due to a misunderstanding of their meaning (Section 1.1), results from this study suggest this applies to only a small proportion of the Southern Asian community surveyed (11%) who incorrectly identified the meaning of the regular flags as indicating an unsafe (10%) or a private swimming area (1%). However, this may not be the case across all multicultural communities [12, 13].

Given the risk factors identified for this community, it is of significant concern that three quarters of the Southern Asian community respondents who had heard of the flags either do not always swim between them, often rarely or never (Table 5). Similarly, just over half acknowledged that they have previously entered the water at an unpatrolled beach, mostly as a family or group (Table 4). These results are higher than those reported for international students at Australian universities by Ballantyne et al. [13] and Clifford et al. [32] and by general beachgoers on beaches in NSW [28]. Clearly, some members of the Southern Asian community are not receiving, understanding, or acting upon the ‘swim between the flags’ message as soon, or as much, as they should be.

The fact that many of the surveyed Southern Asian community were beach safety aware is apparent from almost half stating that the hazard they are most worried about at the beach is drowning or rip currents (Table 4). However, the results of this study have shown that their understanding and recognition of the rip current hazard is poor (Table 4; Fig 4) and less than those of other Australian beachgoers [28]. The Southern Asian community in Australia would benefit from improved education regarding the rip current hazard, including the message that rip currents often appear as seemingly calmer waters.

A significant challenge exists in communicating beach safety information to multicultural communities, particularly as one-in-five respondents had never seen any beach safety information (Fig 5) and more than half did not think the existing amount reaching their community was enough. The majority also thought such information would be more effective if translated into different languages (Table 5). It can take many years for migrants to adjust to life in Australia and become financially, emotionally, and socially ready to participate in beach and water safety programs [53–55]. For this reason, it is likely that receiving this information from within their own community may represent a more successful approach due to higher levels of accessibility, cultural relevance, appropriateness and support [7, 9, 38]. As noted by Scarr et al. [23] a key priority is to identify and engage key leaders from within multicultural communities to champion the benefits of swimming and water safety. However, as evident from results of this study, significant challenges exist in this regard as very few respondents had received or saw the importance of receiving beach safety information from their community (Fig 5).

In order to place potential beach drowning risk factors of the Southern Asian community in more context, a secondary aim of this study was to compare some of the findings from the Southern Asian community with similar results from the overall Australian population. This is possible because Surf Life Saving Australia conducts a national representative online survey each year to capture data about the Australian population’s relationship with the coast [56]. Based on this survey, the average Australian visits the coast 3.3 times a month, 56% always choose to swim between the flags when swimming or wading, 9% indicated they can’t float or swim, and 78% said that rip currents were the most dangerous coastal hazard [5]. In contrast, almost half (48%) of the Southern Asian respondents in this study visit beaches only once a month and 40% visited 1–2 times a year or less. Only 28.5% said that they swim between the flags all the time, 43% indicated they could not swim, and 25% had never heard of a rip current while 24% had, but did not know what they were. Although the Southern Australian community has only made up 5.3% of all coastal drownings in Australia since 2004, these comparisons suggest that the risk of beach drowning amongst this community is a much more serious problem than the overall drowning statistics suggest. This provides further support to the importance of improving beach safety education prioritised for this specific community.

### 4.1. Limitations and suggestions for future study

There were challenges involved in distributing the survey in the priority population. A total of 72 organisations and community groups associated with the community in Australia were contacted directly via email, but only 12 responded (17%) and not all ultimately promoted the survey. Use of social media was more successful, with 37% (n = 12) of community Facebook pages and groups contacted agreeing to promote the survey. The low responses may have been partly due to the survey timing being after the summer season. COVID-19 restrictions also resulted in many multicultural community groups and organisations cancelling programs and events where they might have been able to distribute the survey.

The survey dissemination methods may have potentially biased the sample in some cases. For example, 25% of the surveyed community indicated that they had previously attended a beach/water safety program, but this value may be an overestimate given that some of the surveys were disseminated by organisations directly involved in providing water safety programs to multicultural communities. Also, many of the surveys were from visitors to the SVT temple in southern Sydney who also often visit nearby beaches. This may have potentially biased the sample towards regular temple visitors who may visit beaches more frequently.

While this study focussed on the Southern Asian community in Australia and specific results may not be generalizable beyond the study population, the lessons learned from this work apply to a broad range of communities both in Australia and globally. This study shows the importance of evaluating specific sub-populations within a region: the beach safety knowledge, attitudes, and self-reported behaviour of the Southern Asian community in Australia differed from the national profile. This finding is likely true for other cultural and linguistic sub-populations and has major implications for safety management and future beach safety education programs. The present research provides strong justification for coastal safety organisations and researchers to critically examine language and cultural factors influencing beachgoing behaviour and beach safety knowledge among different communities, providing a foundation from which to prioritise tailored interventions that are culturally and linguistically appropriate.

The importance of culture in conducting a study of this nature was also evident. When surveying predominantly Indian and Nepalese people from the Hindu faith at the Temple, surveyors noted that females were often more hesitant to participate compared to males, both individually and in groups. This was particularly the case in a family setting where females would often defer to their male partners. This male bias was also noticeable from the reluctance of individual females to conduct surveys with male surveyors, although it still occurred with female surveyors in a family context. Females may be reluctant or hesitant to complete the survey because of the gender inequality and patriarchy that is so prevalent in Southern Asia [57]. This partly resulted in the gender imbalance of the survey (Fig 1A).

## 5. Conclusions

This study represents the first evidence-based study in the context of beach safety of multicultural communities in Australia or globally. It has identified a Southern Asian multicultural community at risk of drowning at Australian beaches due to a number of key factors, with those who have spent less than 10 years living in Australia being at most risk. Future swimming and beach safety education programs should prioritise new or recent migrants and increased efforts should be made to assist the development and communication of these programs within multicultural communities themselves. Organisations responsible for beach safety interventions in Australia should also be aware of the importance and need for improved language communication and work with multicultural communities to ensure that future efforts are culturally appropriate. To address the goal of the Australian Water Safety Strategy 2030 to reduce drowning amongst multicultural communities, similar studies should be conducted in partnership with other multicultural communities in Australia.

Finally, this study has shown the importance of understanding beach safety knowledge and behaviour amongst a broader range of communities that to date have been historically excluded, under-served, or marginalised, not just in Australia, but globally. This is particularly true of countries and regions with dynamic and energetic coastlines that have also received a large number of immigrants in recent years, where beach safety education may have been largely overlooked amongst other issues.

## Supporting information

S1 Survey Multicultural beach safety survey. (DOC)

S1 Data Multicultural beach safety survey dataset. (CSV)
