# Supplementary material for: Identifying risk factors and implications for beach drowning prevention amongst an Australian multicultural community
Source: PLoS One. 2022 Jan 11;17(1):e0262175. doi: 10.1371/journal.pone.0262175 (PMC8751996; doi:10.1371/journal.pone.0262175)
Supplement: S1 Survey — (DOC) [file pone.0262175.s001.doc]

**Supplement 1: Multicultural Beach Safety Survey**

Welcome to the UNSW Sydney Survey on the Beach Safety Knowledge of Multicultural Communities

**Q1** Please carefully read the information on the participant information sheet carefully before taking part in the survey.

**Q2** Do you agree with the terms listed in the participant information sheet?

- I agree (Start Survey)
- I don't agree (End Survey Now

**Q3** My First Name is...

________________________________________________________________

**Q4** My email address is...

________________________________________________________________

**Q5** Please answer the following 2 questions to ensure you meet requirements to participate in this survey

**Q6** Are you age 18 years or older? [Please select **one** answer]

- Yes
- No

**Q7** Do you identify as either Indian, Nepalese, Bangladeshi, Sri Lankan, Pakistani, Afghani, Maldivian or Bhutanese? [Please select **one** answer]

- Yes
- No

**Q8** Which multicultural community do you most identify with? [Please select **one** answer]

- Indian
- Nepalese
- Bangladeshi
- Sri Lankan
- Pakistani
- Afghani
- Maldivian
- Bhutanese

|  |
| --- |

Demographics and Beach Visitation

*Now we would like to ask some questions about you and visiting the beach.*

**Q9** How old are you? [Please select **one** answer]

- 18-19
- 20-24
- 25-29
- 30-34
- 35-39
- 40-44
- 45-49
- 50-54
- 55-59
- 60-64
- 65-69
- 70-74
- 75 and over

**Q10** What is your gender? [Please select **one** answer]

- Male
- Female
- Identify as other

|  |
| --- |

**Q11** What country were you born in? [Please select **one** option from the dropdown list]

I was born in _____________________________________________

**Q12** What religion do you most identify with? [Please select **one** answer]

- Atheism / Agnosticism
- Baha'i
- Buddhism
- Christianity
- Hinduism
- Islam
- Jainism
- Judaism
- Kiratism
- Sikhism
- Other (please say below)

________________________________________________

- Prefer not to say

**Q13** What languages do you speak? [Please select **one** answer]

- English only
- Multiple languages, I speak English and... (please list what other languages you speak separated by a space)

________________________________________________________________________

**Q14** How long have you lived in Australia? [Please select **one** answer]

- Less than 1 year
- 1-2 years
- 3-5 years
- 6-10 years
- 11-15 years
- 15-20 years
- More than 20 years
- All of my life

**Q15** What is your home postcode [Please select **one** answer]

- My home postcode is ________________________________________________
- Unsure

**Q16** How often do you visit an ocean beach? [Please select **one** answer]

- Daily or weekly
- Monthly
- 1-2 times per year
- Less than 1 time per year
- Never (Please Skip to Q19)
- Unsure

**Q17** How often do you go in the water when you visit an ocean beach? [Please select **one** answer]

- Never
- Rarely
- Some of the time
- Most of the time
- Every time
- Unsure

**Q18** What are the main things you do when you visit an ocean beach? Please rank from 1 to 3, with 1 being the thing you do the most [You must rank at **least 1** option]

______ Swimming

______ Playing in the water

______ Lying in the sun / Sunbathing

______ Picnics/parties/family events

______ Religious events

______ Walking/jogging

______ Fishing

______ Surfing/bodyboarding

______ Kayak/canoe

______ Other

Swimming Ability and Beach Safety Behaviour

*Now we will ask some questions about your swimming ability and knowledge of beach safety.*

**Q19** Can you swim? [Please select **one** answer]

- Yes
- No (Please skip to Q24)
- Unsure

**Q20** How far can you swim in a swimming pool? [Please select **one** answer]

- Less than 25 metres without stopping
- Up to 100 metres without stopping
- More than 100 metres without stopping
- More than 500 metres without stopping
- Unsure

**Q21** Have you ever been swimming / bathing in the ocean before? [Please select **one** answer]

- Yes
- No (*Please skip to Q25*)
- Unsure

**Q22** How far can you swim in the ocean? [Please select **one** answer]

- Less than 25 metres without stopping
- Up to 100 metres without stopping
- More than 100 metres without stopping
- More than 500 metres without stopping
- Unsure

**Q23** Are you confident swimming in the ocean when there are waves? [Please select **one** answer]

- Yes, but only with small waves
- Yes, with both small and large waves
- No
- Unsure

**Q24** When you go in the water at an ocean beach, how often do you enter fully clothed? [Please select **one** answer]

- Never
- Rarely
- Some of the time
- Most of the time
- All of the time
- Unsure

**Q25** Have you ever had organised / formal swimming lessons before? [Please select **one** answer]

- Yes (*Please skip to Q27*)
- No (*Please answer Q26*)
- Unsure

**Q26** Are there any reasons why you haven't had organised / formal swimming lessons before? [Please select **any** that apply] (*Answer this question and skip to Q29*)

- Lack of time
- Financial cost
- Lack of availability
- Other ________________________________________________

**Q27** Where have you had these organised / formal swimming lessons? [Please select **any** that apply]

- In Australia
- In another country (please write the name of country in the box, multiple countries separated by a space)

________________________________________________

- Unsure

**Q28** When have you had these organised / formal swimming lessons? [Please select **any** that apply]

- As a child < 12 Years
- As a teenager 13-17 years
- As an adult > 18 years
- Unsure

**Q29** Below is a list of factors related to visiting the beach. Please answer how important each factor is to you [Please answer **all** responses]

|  | Unsure | Not important | No feeling either way | Important |
| --- | --- | --- | --- | --- |
| Clean beach and water |  |  |  |  |
| Lots of people around |  |  |  |  |
| Not many people around |  |  |  |  |
| Calm water |  |  |  |  |
| Big waves |  |  |  |  |
| Beach with lifeguards/lifesavers |  |  |  |  |
| It is close to home |  |  |  |  |
| Easy parking |  |  |  |  |
| Getting there using public transport |  |  |  |  |
| Beach is safe for swimming |  |  |  |  |
| Space for picnics and large gatherings |  |  |  |  |

**Q30** Please look at each of the photographs below. 

In each of the photos, place an ‘**X**’ on the location where you would enter the water to swim, wade or play. If you would not enter the water, please select *‘I would not swim’*.

**Photo 30A**


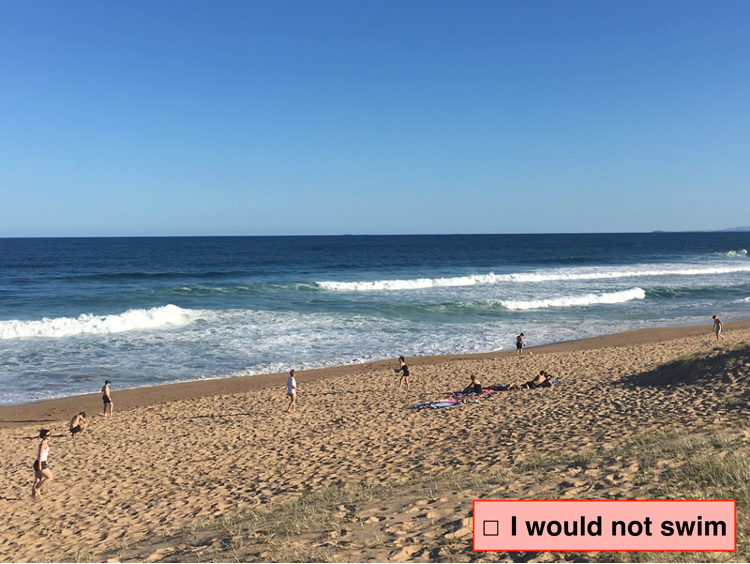


**Photo 30B**


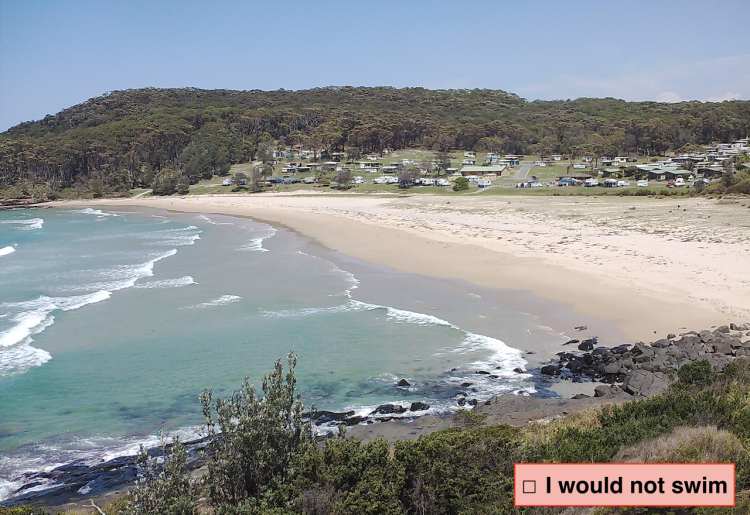


**Q31** When you visit the beach, how often do you look for dangers? [Please select **one** answer]

- Never
- Rarely
- Some of the time
- Most of the time
- All the time
- Unsure

**Q32** What are you worried about the most when you visit the beach? [Please select **one** answer]

- Sunburn
- Jellyfish / bluebottles
- Sharks
- Waves
- Rip currents
- Drowning
- Lots of people
- Dirty water / beach
- People stealing my things
- Other ________________________________________________
- Nothing
- Unsure

**Q33** Have you heard of the red and yellow beach flags on Australian beaches? [Please select **one** answer]

- Yes
- No (*Please skip to Q37*)
- Unsure

**Q34** What is the meaning of the red and yellow flags? [Please select **one** answer]

- The area between the red and yellow flags is a private swimming area
- The area between the flags marks a safer area of the beach for everyone to swim
- The area between the flags marks an area unsafe for swimming
- Other ________________________________________________
- Unsure

**Q35** If you go in the water on a beach with red and yellow flags, how often do you swim between the flags? [Please select **one** answer]

- Never
- Rarely
- Some of the time
- Most of the time
- All of the time
- I do not swim at the beach
- Unsure

**Q36** Have you ever entered the water at a beach **with no** red and yellow flags? [Please select **one** answer]

- Yes, mostly by myself
- Yes, mostly with family or a group of people
- No
- Unsure

**Q37** Do you know what a rip current is? [Please select **one** answer]

- No, I have never heard of a rip current (*Please skip to Q39*)
- I have heard of a rip current, but I don't know what it is (*Please skip to Q39*)
- Yes, I know what a rip current is
- Unsure

**Q38** Please look at each of the photographs below. 
 
If you do not think there is a rip current in the photos, please **place an ‘X’** on the ‘No Rip’ box. 
 
If you think that there is a rip current in the photos, please **place an ‘X’** on the area where you think a rip current is located.

**Photo 38A**


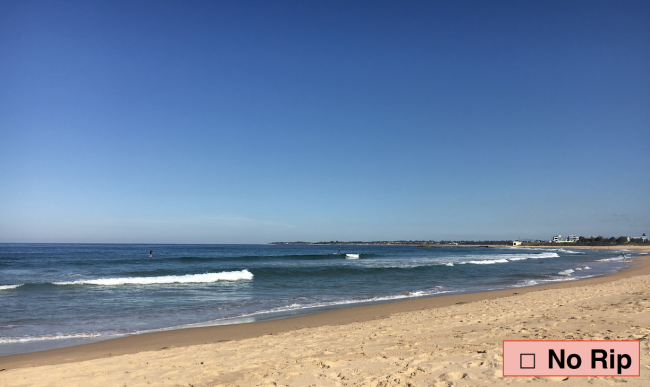


**Photo 38B**


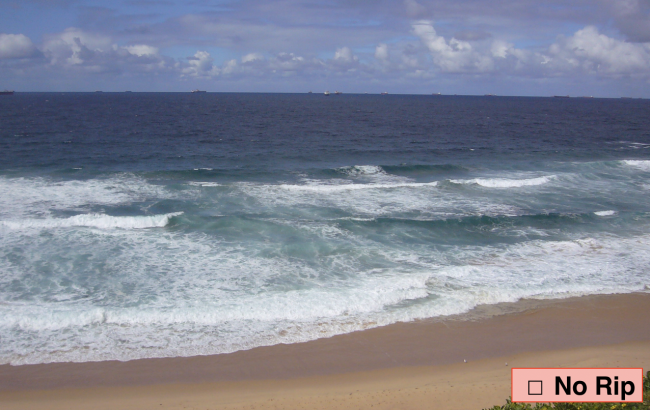


**Q39** Have you ever attended a beach or water safety education program or presentation? [You may select more than**one** answer]

- No
- Yes, at a primary school
- Yes, at a high school
- Yes, at university as an international student
- Yes, in my community
- Yes, at a surf lifesaving club
- Yes, other (please indicate)...________________________________________
- Unsure

**Q40** Where have you seen or heard information about beach safety? [Please select **any** that apply]

- I have never seen or heard information about beach safety before
- Social media (for example Facebook, Instagram, Twitter)
- Online (Internet websites and Youtube videos)
- Surf Lifesaving Beachsafe App
- TV / Radio
- Newspapers (printed or online)
- Signs at the beach
- At a primary school
- At a high school
- At a university
- From lifeguards / lifesavers
- From friends or family
- From my own community
- Other ________________________________________________
- Unsure

**Q41** Do you think there is enough beach safety education reaching you and your community? [Please select one answer]

- I don't think beach safety information is important
- The present amount is good
- The present amount is not good, there needs to be more
- Unsure

**Q42** What is the main way you would like to receive beach safety information in the future? [Please select **one** answer]

- Social media (for example, Facebook, Instagram or Twitter)
- Online (for example, Internet websites or Youtube videos)
- Surf Lifesaving Beachsafe App
- TV / Radio
- Newspapers (printed or online)
- Signs at the beach
- At a primary school
- At a high school
- At a university
- From lifeguards / lifesavers
- From friends or family
- From my own community
- I don't want to receive beach safety information in the future
- Other ________________________________________________
- Unsure

**Q43** Do you think beach safety information would be more effective in multiple languages? [Please select **one** answer]

- Yes, it needs to be translated into more languages
- No
- Unsure

**Q44** Please enter your email if you would like to participate in a future focus group discussion about beach safety

- My email is... ________________________________________________
- I do not want to participate in this future event

**End of Survey**

Thank you for participating in the UNSW Beach Safety Survey on the Beach Safety Knowledge of Multicultural Communities
